# Regulating algorithmic care in the European Union: evolving doctor–patient models through the Artificial Intelligence Act (AI-Act) and the liability directives

**DOI:** 10.1093/medlaw/fwae033

**Published:** 2024-09-10

**Authors:** Barry Solaiman, Abeer Malik

**Affiliations:** HBKU Law, Qatar; Weill Cornell Medicine, Qatar; HBKU Office of the Vice President for Research, Qatar

**Keywords:** artificial intelligence (AI), artificial intelligence act (AI-Act), doctor–patient relationship, European Union, health care, directives

## Abstract

This article argues that the integration of artificial intelligence (AI) into healthcare, particularly under the European Union’s Artificial Intelligence Act (AI-Act), poses significant implications for the doctor–patient relationship. While historically paternalistic, Western medicine now emphasises patient autonomy within a consumeristic paradigm, aided by technological advancements. However, hospitals worldwide are adopting AI more rapidly than before, potentially reshaping patient care dynamics. Three potential pathways emerge: enhanced patient autonomy, increased doctor control via AI, or disempowerment of both parties as decision-making shifts to private entities. This article contends that without addressing flaws in the AI-Act’s risk-based approach, private entities could be empowered at the expense of patient autonomy. While proposed directives like the AI Liability Directive (AILD) and the revised Directive on Liability for Defective Products (revised PLD) aim to mitigate risks, they may not address the limitations of the AI-Act. Caution must be exercised in the future interpretation of the emerging regulatory architecture to protect patient autonomy and to preserve the central role of healthcare professionals in the care of their patients.

## I. INTRODUCTION

This article explores the potential transformations in the doctor–patient dynamic as artificial intelligence (AI) becomes increasingly integrated into healthcare practices, particularly under the regulatory framework proposed by the European Union’s (EU) Artificial Intelligence Act (AI-Act). A paternal relationship historically governed doctors and patients in Western medical practice but gradually receded in the name of patient autonomy and its corollary consumeristic practices.^1^ In its place, a business-to-consumer (B2C) environment has provided an opportunity to reconcile patient empowerment and equity.^2^ That empowerment has arisen partly because of technology. Patients can access a plethora of information online, health devices are becoming smaller and more powerful, and the physical space that healthcare professionals once occupied is shifting online through telemedicine.^3^ While technological advancements have been ubiquitous in medicine, digitisation has led to rapid developments.

AI is increasingly used to power medical devices, giving them powerful predictive and analytical capabilities that can surpass healthcare professionals while saving time and costs. For these reasons, hospitals worldwide are deploying AI, with some possessing dedicated AI departments.^4^ The rise of ChatGPT and Generative AI (GenAI) highlights how the use cases will continue to expand, and AI will take on an increasingly prominent role over time. As such, it is crucial to examine how AI might intersect with the doctor–patient relationship, particularly within the rubric of the EU’s AI-Act, the first comprehensive law on regulating AI in the world.^5^ The future signals different potential pathways. Patients may gain more autonomy over their health care, doctors may gain more power over medical decision-making by using AI systems (thus re-entrenching paternalism), or both doctors and patients may be disempowered as decision-making is ceded to private entities. While the proposed AI Liability Directive (AILD) and the Directive on Liability for Defective Products (PLD) may help to reduce the scale of patient disempowerment, much remains unclear about their potential impact.

To examine these pathways, this article is divided into three parts. First, there is an analysis of the doctor–patient relationship as it has evolved in recent decades. Secondly, this article examines different doctor–patient models that exist today and evaluates how AI intersects with those models. Part three evaluates the limitations of the AI-Act, arguing that the risk-based approach threatens to disempower both doctors and patients by failing to protect the end user (the patient) on the front end. While the proposed liability directives may offer some avenues for redress on the back end, they apply too late once harm has arisen and are, in any case, likely to be limited in their application. If the flaws are not addressed, the regulatory system could shift powers to private entities, undoing patient autonomy.

## II. THE EVOLVING DOCTOR–PATIENT RELATIONSHIP

While practice will vary amongst doctors, the dominant paradigm of the relationship between doctors and patients was historically paternalistic. The doctor was the dominant party, and the patient was there to answer questions and follow orders.^6^ Patients could not expect adequate explanations regarding their treatment or (to avoid distressing patients) important information about their health was not shared.^7^ Those practices are generally considered unethical today unless a patient lacks mental capacity, is unconscious, or is a child. In those cases, paternalism may be acceptable in principle and necessary in practice because those individuals are incapable of making decisions. The decision of what to do in each case will depend on the patient’s best interests.^8^

Those cases aside, there has been a move away from paternalism encouraged by court cases and the guidance of professional bodies. Patients are encouraged to ask questions, and doctors might assume patients have some knowledge because of the Internet.^9^ The UK Supreme Court in *Montgomery v Lanarkshire Health Board*^10^ has noted the shift from paternalism towards a system that treats patients ‘as adults who are capable of understanding that medical treatment is uncertain of success and may involve risks, accepting responsibility for taking risks affecting their own lives, and living with the consequences of their choices’. The General Medical Council emphasises this approach, noting that doctors should work in partnership with patients by listening to their concerns, providing the information they want, respecting their decisions, and supporting them in caring for themselves.^11^

This emphasis on ‘patient autonomy’ has arisen at a time of a converging culture of consumerism across jurisdictions. This is apparent when examining the ‘consumer’ trend globally. In the USA, patients were first described as ‘consumers’ by medical economists in the 1930s.^12^ Though, it was not until the 1960s that the concept began to gain mainstream acceptance.^13^ In Europe, the marketisation of healthcare arose more prominently in the 1980s.^14^ Those changes coincided with a growing Kantian atomistic concept of individual autonomy premised on self-governance and self-reliance.^15^ The motivation was to protect individuals from the harsher elements of paternalism, which pit the naïve patient against the sophisticated healthcare professional possessing expertise and unequal bargaining power—a ‘David-versus-Goliath’ dynamic.^16^

Over time, there was a growing awareness of the importance of patient rights.^17^ There were many patient organisations through the decades, but their underlying philosophy was that the ‘patient/consumer must be the central concern of healthcare’.^18^ Today, the ‘patient-consumer’ framework can be found to some degree in every EU Member State’s healthcare system and is considered a key theme of EU health law.^19^ This move towards an individualistic and consumeristic model is perhaps best articulated in the UK Supreme Court case of *Montgomery v Lanarkshire Health Board* in 2015 when it was noted that:

Patients are now widely regarded as persons holding rights, rather than as the passive recipients of the care of the medical profession. They are also widely treated as consumers exercising choices: a viewpoint which has underpinned some of the developments in the provision of healthcare services.^20^

This consumeristic dynamic is captured most acutely in the transnational context through medical tourism or the free movement of patients in the EU.^21^ Indeed, Pattinson notes how the study of medical law cannot ‘ignore the developing consumerism of patients, the associated medical tourism and the impact of market forces’.^22^ In medical tourism, ‘commodification, consumerism and care go hand-in-hand’.^23^ Patients are not only holders of rights but are purchasing a commoditized service. A private market encourages a ‘buyer-seller relationship’. That paradigm is an extreme manifestation of consumerism, but it illustrates how patients have gained autonomy in the last century and have become consumers. The query is how these changes have influenced the doctor–patient relationship and what that means when AI is incorporated.

## III. PINPOINTING THE DOCTOR–PATIENT RELATIONSHIP TODAY

To elucidate the implications of AI, it is first helpful to highlight different models of the doctor–patient relationship. The traditional paternalistic approach is explained by the ‘physician as parent’ model, in which the metaphorical ‘benevolent father’ determines the best interests of the ‘ignorant child’.^24^ The patient is subordinate to the doctor whose recommendations they follow.^25^ That model jars with today’s consumeristic or ‘contractualism’ trend that other models detail.^26^

A second model is the ‘physician as partner’ that emphasises equal power, open deliberation, and a symmetric flow of information between the doctor and the patient.^27^ A third model is the ‘physician as (service) provider’ reflecting the consumerism that arose in the USA in the 1960s and the involvement of private elements in European healthcare systems from the 1980s.^28^ The ‘service provider’ model differs from the ‘partner’ model in terms of patient power and autonomy.^29^ For the ‘service provider’, the doctor is the seller, the patient is the buyer, and the power rests with the patient who makes the purchasing decision.^30^ Instead of being trusted, the physician is held to account and supervised. Only the patient is genuinely interested in their health.^31^ In practice, the trend was towards both these models in the twentieth century, with the doctor and patient sharing responsibilities consensually.^32^ The educated consumer–patient might have more meaningful relationships with their doctors, and doctors may be more willing to share control over a patient’s health management with patients who communicate well.^33^

Shutzberg proposes a fourth model, the ‘equal disempowerment of physicians and patients’, which is pertinent to the analysis of the AI-Act below.^34^ The partnership and consumer models assume that power is concentrated between the patient and doctor with different degrees of distribution. Yet, in practice, that power has flowed to external market forces encouraged by neoliberalism.^35^ Shutzberg argues that this dynamic of equal disempowerment has become paradigmatic.^36^ Individualism is promoted as patient choice, with the private sector gaining more responsibility and citizens having less dependency on the State.^37^ This creates greater social and medical inequality because socio-economic background becomes an increasingly important factor in an individual’s health outcomes.^38^ Those with greater financial resources have more choices.

There is also possibly a fifth ‘technology’ model. Garrot and Angele-Halgand discuss the ‘emergence of a new relationship between the patient and his/her health’ through technology.^39^ Cumming and others similarly discuss an ‘integrated and participatory approach’ premised on requiring ‘the full engagement of people in their own health-care and lifestyle decisions’.^40^ This is similar to the biopsychosocial model (BPS) from the 1970s.^41^ The BPS model emphasises that the individual is the only person who can effect behavioural change (perhaps with help from friends) and that there are cases where professional assistance is needed.^42^ Where those cases arise, a blueprint for effective assistance should be created to help the individual.^43^ Emanuel and Emanuel also discuss the ‘instrumental model’, which may fall within the technology model. Here, the doctor disregards patient values and relies on external values, like social or scientific benefit, for treatment decisions. While morally condemned, the model bears relevance to AI as the doctor–patient relationship risks being ‘instrumentalized’ where AI is used ‘not for the good of the patient, but rather for the sake of efficiency or cost savings’.^44^

These models will play out in different ways through the interactions between doctors and patients and some will dominate at different times. However, of these models, the proliferation of AI will likely increasingly implicate the fourth and fifth models and their associated impact on patient autonomy.

### A. Technology and the doctor–patient relationship

Technology is causing a profound shift by reinforcing patient autonomy. It is commonplace for people to look up health information online.^45^ mHealth apps have proliferated, and individuals increasingly use wearables to monitor their health^46^ Technology empowers and motivates individuals to manage their health on the move, with the focus shifting towards prevention.^47^ In some cases, technology is integrated into the doctor–patient relationship, with the care provider using devices to monitor patients remotely.^48^ Smartphones enable physicians to access patient data easily.^49^

Existing models can explain this technological paradigm. If doctors continuously track their patients remotely, this could reflect a heightened paternalistic approach, whereby the doctor oversees an individual’s health in a manner not possible before. However, the point of such technology is to empower patients to make decisions and be proactive with the information given. More likely, the use of technology is embedded in other doctor–patient models. Technology allows for a continuous symmetric flow of information between the doctor and patient, and it is agreed that a deliberative approach will be taken to healthcare decisions.^50^ Patients also have more autonomy. The doctor provides the device, the device provides information, and the patient decides what to do next. This highlights the digital participation of citizens in their healthcare and a shared decision-making model.^51^

Despite the potential benefits, there is concern that technology puts too much onus on patients.^52^ ‘Empowerment’ risks ‘excessive responsibilization’, so a patient’s healthcare ‘may be more and more incumbent on them’.^53^ This could lower standards and result in a lower quality of care.^54^ Patients may be less likely to seek care from a professional, instead choosing to ‘self-diagnose, self-medicate and self-experiment’—the emergence of the ‘self-patient’.^55^ The extent of this risk is both unclear and hard to control.^56^ Patients might take unlicensed medications without professional oversight, undermining the doctor–patient relationship owing to that ‘responsibilization’.^57^ The ‘self-patient’ may promote a bottom-up approach, challenging evidence-based medicine premised on applying rigorous standards and methodologies. There may also be an ‘extreme’ scenario where patient health monitoring becomes the norm ‘to such an extent that it becomes less the voluntary act of an engaged citizen than a progressively obligatory requirement on individuals’.^58^ The challenge is to strike a balance between patients exercising their autonomy while maintaining the interpersonal exchange and expert support of doctors.^59^

From the doctor’s perspective, technologically mediated care may also limit their contextual understanding of the patient. The reliance on data from technology risks overlooking essential contextual factors, such as mental health and emotional states, hindering the development of trust between doctors and patients and limiting opportunities for clinicians to incorporate tacit knowledge into patient care.^60^ Another dimension is patient expectations. Technology may raise expectations so high that dissatisfaction and disillusionment may ensue where they are not met. The ensuing distrust and uncertainty ‘may form at least part of the backdrop to citizen involvement in medicine’, the ‘expert patient’ and ‘even the trend of defensive medicine’.^61^ Instead, technology should enhance the doctor–patient relationship and the quality and availability of healthcare provision.

Technology can, therefore, have a dichotomous impact on care depending on whether it is used as a passive information tool or more proactively. Nowadays, technology is evolving to a level that usurps human medical decision-making. The query is how AI systems might impact the relationships examined so far.

### B. AI systems and new patient models

The uses of AI in healthcare have been exhaustively examined in the literature and will not be revisited in detail here.^62^ Presently, AI systems act primarily as a decision support tool for doctors. For example, in radiology, AI analyses images for cancer, trauma, Alzheimer’s disease, coronary artery disease, pneumonia, and haemorrhages to identify, classify, and predict medical issues and can do so more accurately than doctors.^63^ GenAI systems (such as ChatGPT) can analyse information in an electronic health record (EHR) and detect diseases early.^64^ Patients can be tracked remotely using wearable AI (such as smartwatches) to analyse vital signs and identify and predict health problems.^65^ All such uses of AI require a healthcare professional’s oversight and final decision. Yet, AI’s sophistication might shift responsibilities in the doctor–patient relationship.^66^

As with other technologies, AI may entrench paternalism when used as a tool for monitoring and control. Nevertheless, AI is more likely to implicate the ‘physician as partner’ or the ‘physician as (service) provider’ models, with the doctor and patient considering the recommendations of an AI system in a back-and-forth discussion over the best options for the patient’s treatment. In those circumstances, AI can enhance autonomy by supporting informed decision-making. AI systems also arguably highlight a shift towards other models of patient care, as noted earlier. First, the ‘technology model’,^67^ which might require people’s full participation and engagement in their healthcare decisions or lead to physicians’ overreliance on AI.^68^ Secondly, the ‘equal disempowerment of physicians and patients’ to external market forces.^69^

Under the technology model, patients must get used to AI being part of their care in the clinic or at home. This requires patients to be actively engaged when the doctor is not present and to make decisions about their care. In this model, autonomy may widen in scope because responsibilities typically preserved for the doctor may shift towards patients. That is good in certain scenarios. For example, AI could be more ‘ethical’ than a doctor because a system could provide unbiased information that is not prone to human fallibility.^70^ Indeed, it has been emphasised how an AI system that ‘values’ patients can be a ‘significant aspect of a trusting relationship’. ^71^ Shared human values may be of more import than programming parameters.^72^ However, that is easier said than done. AI systems may not replicate a doctor’s empathetic ear and words, and while the technology empowers patients with greater access to medical information, potentially misleading or inaccurate information may diminish its effectiveness.^73^ Patients may derive autonomy, but AI may not uphold their dignity like a human relationship.^74^ Further, if patients are obliged to act on AI recommendations, they could be held responsible if they fail to do so and harm occurs.^75^ Thus, there is concern about the appropriate limits on patient autonomy and the acceptable latitude given to healthcare providers to devolve decision-making. Furthermore, autonomy may not be enhanced where AI instrumentalizes the doctor–patient relationship so that the doctor overly relies on AI recommendations without sufficient rigour.

These are pertinent considerations that future research must examine. For the examination of the AI-Act, the latter model on the disempowerment of doctors and patients to market forces is most relevant for several reasons. Firstly, data privacy is a significant consideration because vast amounts of data are processed to train AI systems and examine patient health, threatening confidentiality in the doctor–patient relationship.^76^ Patients must be informed about how their data are processed and be protected from harm.^77^ Patient data can be misplaced and reidentified despite being anonymised, which raises General Data Protection Regulation (GDPR) considerations.^78^

Secondly, the opacity of AI systems poses a threat to patient autonomy. Algorithms are so complex that finding the ‘reasoning’ for a decision is almost impossible, even for computer scientists. Developers are creating algorithms that offer ‘explainability’ for their decisions, but the technology is far short of replicating complicated doctor–patient interactions. This could cause patients to lose some understanding and control over their care.^79^ Information is essential for patients to provide informed consent (one of the cornerstone protections of medical law) but it is unclear how transparent AI systems can be, nor what the appropriate level of information should be.^80^ As discussed earlier, AI systems utilise extensive data and intricate statistical techniques in decision-making, posing challenges to understanding the full extent of data processing that informs one’s diagnosis and treatment.^81^ It is also worth noting that in instances where AI systems offer clinical expertise, such as diagnosing a condition or interpreting scans, the responsibility to explain one’s decision-making seemingly shifts from ‘doctor to AI system, or at least to manufacturer of AI system’.^82^ At present, analyses of existing case law in the USA suggest that doctors will generally not be liable for failing to inform patients about considering AI recommendations for their care, but it is unclear whether that will be the case in the EU.^83^ Further, private companies will continue to develop and profit from medical AI, which could deplete autonomy until explainability is rectified and the legal rubrics for protecting informed consent are adequately developed.

Thirdly, even if doctors do not become reliant on AI, accountability concerns arise for patients harmed by AI recommendations. Drawing the line of responsibility between regulators, healthcare providers, and AI developers is complicated, and the answer may shift over time.^84^ While healthcare providers are liable for their negligence, following AI advice may become incorporated into the standard of care moving forward.^85^ Questions remain over how departing from an AI recommendation would fall below the standard of care, but the future could lead to equal disempowerment if market forces are left to create AI products that determine the appropriate standard of care.

Fourthly, doctors could become over-reliant on AI, leading to a gradual decline in their skills and the personal connection between doctors and patients.^86^ Owing to this void, developers may gain too much influence over healthcare, but AI may be unable to fill the human void. If AI cannot comprehend patients’ views, it will not replicate the doctor–patient relationship.^87^ The values that underpin AI decisions may not comport with those of patients, such as systems that value decisions maximising one’s lifespan rather than minimising one’s suffering.^88^ Consequently, developers must use their power responsibly to influence public views and ensure that their systems are safe and helpful to patients.^89^

Much of the preceding analysis might indicate a future focused on developer liability. Perhaps one can be sanguine about autonomy because patients could sue the developer in a hyper-commercial reality that pits the patient–consumer against market forces now imbued with caregiving powers through their AI systems—the hospitals merely existing as the vassal through which those forces coalesce. Some devices are subject to regulatory control through the Food and Drug Administration (FDA) in the USA or regulations on medical devices in Europe (the limitations of the latter are noted below). However, jurisprudence in the USA suggests that developers gaining FDA approval will be shielded from tort law liability in some cases but not in others, depending on the approval pathway followed.^90^ Much is still to be determined, so it is unclear what avenues for redress will be available for patients.

AI raises concerns about data privacy, explainability, informed consent, medical liability, and an over-reliance on technology. While the doctor–patient relationship has shifted to one emphasising autonomy, AI could disempower both groups. Problematically, the EU’s new legal infrastructure may not do enough to protect against these risks.

## IV. THE AI-ACT’S RISK-BASED APPROACH AND ITS FLAWS

While the EU recognises that AI has benefits and risks for health, the AI-Act is more general in scope. It creates a rigid approach to classifying risk that focuses on systems and processes rather than substantive rights.^91^ The law covers companies instead of individuals most vulnerable to the effects of AI systems.^92^ It has been argued that ‘this is especially harmful in the clinical context, as patients are particularly susceptible to the risks of AI because of the inherent dependency and information asymmetries in the patient–health professional relationship’.^93^ The Commission wants to balance encouraging innovation while respecting the EU’s values.^94^ This approach raises questions about its symbolic meaning for the doctor–patient relationship. The AI-Act has ‘severe weaknesses’ in its comprehensiveness and clarity, consisting of a patchwork of existing regulations, fundamental rights protections, and consumer protection laws.^95^ These concerns can be seen when deciphering the Act’s risk-based approach.

Under the regulation, AI systems fall into one of three categories. First, AI that poses an ‘unacceptable risk’ is prohibited. Second, ‘high-risk’ AI systems posing a significant harmful impact on the health, safety and fundamental rights of persons must comply with certain requirements and procedures. Third, low-risk AI is subject only to minimum transparency requirements.^96^ The AI-Act refers broadly to ‘AI systems’ throughout, meaning machine-based systems designed to operate autonomously, that may exhibit adaptiveness after deployment, and that, for explicit or implicit objectives, based on input data, infer how to produce outputs such as content, predictions, recommendations, or decisions, influencing the physical and virtual environments with which the system interacts.^97^ The regulation also refers to ‘providers’ and ‘deployers’ throughout. The provider is the person or entity that develops AI and places it ‘on the market or puts the system into service under its own name or trademark’.^98^ The deployer is a natural or legal person, including a public authority, agency, or other body under whose authority the system is used.^99^

Many of the standards created in the AI-Act remain unclear and will be subject to interpretation. Ultimately, the concern is that the regulation does little to anticipate the challenges noted above. The ‘unacceptable risk’ category is unlikely to apply to the doctor-patient relationship because it covers devices that deploy subliminal or purposefully manipulative or deceptive techniques aimed at materially distorting one’s behaviour by impairing their ability to make an informed decision.^100^ As such, this category is not considered here. The main considerations for doctors and patients arise in the ‘high-risk’ category, but it is important to note some of the limitations of the low-risk category first. For low-risk AI systems, there are only minimum transparency requirements.^101^ Providers and deployers of AI systems must inform natural persons that they are interacting with an AI system unless it is ‘obvious from the point of view of a … reasonably well-informed, observant and circumspect’ natural person, taking into account the circumstances and the context of use.^102^ Natural persons should be informed if they are exposed to emotion recognition systems or biometric categorisation systems.^103^ Providers can create codes of conduct that encompass the requirements of the AI-Act, but this will not be monitored, which means that such codes are unlikely to have any practical effect.^104^

One lingering question about the low-risk category is the extent to which providers can categorise their systems as low risk and thus avoid oversight in the healthcare context. In the high-risk category considered below, medical devices are included under the annexes of the AI-Act. Although, the high-risk category also includes AI systems used for public assistance and benefits, including healthcare services, and those used to establish priority in emergency healthcare patient triage systems, the scope remains limited.^105^ Thus, devices such as personal digital assistants that may provide medical advice are not captured.^106^ This is despite the significant risks that AI systems may pose for patients. It has been deduced that AI systems that are not covered by the annexes for medical devices for high-risk systems will presumably be covered by the low-risk categorisation that yields minimum regulation.^107^ This is especially problematic because medical device regulations do not cover all types of AI applications in healthcare, and public health applications that proliferated since COVID-19 may also not be covered. Owing to the enormity of that data and the effects of any disclosure, this presents particular vulnerabilities with data protection.^108^ There is also concern about the safety of mobile apps that pose a clinical risk to consumers, for which recourse is unavailable where harm arises.^109^ The AI-Act appears to do little to protect patients if such systems are recommended for use by doctors.

### A. Deficiencies of the ‘high-risk’ category

The most substantive category under the AI-Act is Chapter III on AI systems that pose a ‘high-risk’ to health, safety or fundamental rights of individuals. In the patient context, AI may threaten core patient rights and the associated principles of autonomy, human dignity, and trust.^110^ AI systems will be permitted into the European market following certain requirements and assessments, with the ‘intended purpose’ forming an important consideration in the classification process.^111^ Two categories of AI systems are envisaged. The first consists of systems covered by the Union harmonisation legislation (listed in Annex II) required to undergo third-party conformity assessments with a view to being placed on the market or being put into service.^112^ The second are systems intended to be used as a safety component of a product covered by the Union harmonisation legislation.^113^

To determine whether an AI system poses a risk, several factors shall be considered by the Commission. Among those factors are:

- the intended purpose of the AI system;
- the extent to which the AI system has been used or is likely to be used;
- the nature and amount of the data processed and used by the AI system, in particular whether special categories of personal data are processed (such as sensitive health data);
- the extent to which the AI system acts autonomously and the possibility for a human to override a decision or recommendations that may lead to potential harm;
- the extent to which the AI system has already caused harm concerning health and safety or fundamental rights or has raised concerns about the likelihood of such harm;
- the potential extent of such harm, particularly in terms of its intensity and its ability to affect a plurality of persons or to disproportionately affect a particular group of persons;
- the extent to which those harmed are dependent on the outcome produced by an AI system and they cannot opt-out from that outcome;
- the extent to which a person is in a vulnerable position or may be adversely affected in relation to the user of the AI system (eg, where there are imbalances in power, economic or social circumstances, knowledge, or age);
- the extent to which the outcome produced by an AI system is not easily reversible (outcomes that have an impact on the health or safety of individual will not be considered easily reversible);
- the extent to which existing Union legislation already provides a remedy.^114^

While much is still to be determined about the scope of these factors in practice, one could envisage their application to AI systems used in healthcare. For example, where a hospital uses an AI system for diagnosis and a patient cannot opt out of its use because it is the only device used by the hospital for such a diagnosis, or where a doctor relies on an AI recommendation to provide treatment that causes harm (an unreversible outcome). One can only posit such cases because many other provisions and processes outlined in the AI-Act are vague or not sufficiently robust, as seen with the compliance measures.

### B. Disempowering compliance mechanisms

One concern highlighted in this article about using AI in health is its potential to disempower doctors and patients. The regulatory schema of the AI-Act could contribute to this disempowerment rather than prevent it. A core limitation of the AI-Act is its heavy—and in some cases, exclusive—reliance on self-conformity assessments to determine whether there is compliance with fundamental rights.^115^ Before high-risk AI systems are permitted to enter the market, they must be granted European conformity (CE) marking.^116^ However, the CE certification system provides weak protection for human health and safety, as evidenced under other related regimes, such as the Regulation on in vitro diagnostic medical devices (IVD), in which fraud and corruption in the industry occurred undetected for a significant time.^117^ Ultimately, it has been argued that ‘it is seriously questionable whether reliance on self-certification provides meaningful legal assurance that the requirements to obtain a CE mark in relation to ‘high-risk’ AI systems are properly met’.^118^

Another problem is that the providers undertake conformity assessments.^119^ The system is essentially ‘trusting providers to police themselves’.^120^ In some other cases, notified bodies verify the conformity of AI systems.^121^ Yet, even in those cases, the same problems of independence may arise. The AI-Act states that notified bodies must be independent of the provider of the AI system, any other operator having an economic interest in the high-risk AI system, and any competitors of the provider.^122^ However, notified bodies cannot always be fully independent because they are usually private sector firms that provide certification.^123^ While such bodies are listed online and subject to certain standards, very little is known about their day-to-day activities or working procedures.^124^ Further, their certification process will primarily focus on the narrow provisions of the AI-Act without deeper consideration of fundamental rights requirements, causing compliance to be reduced to a box-ticking exercise.^125^ Potentially biased private actors who lack expertise will decide whether AI systems follow the law and their subsequent entry into the market, which is ultimately very concerning for patient autonomy. ^126^

Such conformity mechanisms paint a picture of disempowerment for hospitals and patients. The flaws existing in current mechanisms will be replicated with private entities (potentially lacking independence) determining the compliance of AI systems. The entire process threatens to ignore the autonomy of the consumer–patient because fundamental rights are unlikely to be a primary consideration for those entities.

### C. Monitoring through disempowered ‘deployers’

Once an AI system is on the market, the next issue is the continuing conformity of the AI system under the AI-Act. In this regard, many provisions under the AI-Act *could* mitigate some of the risks of AI systems once in use, but may not do so in practice.

The AI-Act requires a quality management system consisting of a strategy for regulatory compliance, systems and procedures for data management, and procedures relating to reporting serious incidents, among others.^127^ The latter requirements concern post-market monitoring of high-risk AI systems. Monitoring should be established by the provider so that they can collect, document, and analyse the relevant data provided by deployers or other sources throughout the lifetime of the AI system to evaluate their compliance.^128^ The concern with this approach is its overreliance on ‘deployers’ for reporting.

‘Deployers’ are defined as ‘any natural or legal person, public authority, agency, or other body using an AI system under its authority’.^129^ Patients do not fall within this definition of a deployer. Presumably, if an AI system is given to a patient under the authority of a hospital, then the hospital (and its healthcare professionals using the AI system) will be defined as a deployer under the regulation. That would accord with the current lines of responsibility whereby doctors are responsible for their patients (and the hospital is vicariously liable for harm). Yet, if the health paradigm were to shift in the future, with the emphasis placed on patients using AI for their own care, then the AI-Act would neglect patients’ rights. Even in their current incarnation, the provisions on providers and deployers are inherently problematic from the hospital provider and doctor’s perspective.

One problem is the flow of information between the provider, deployer, and patient. The AI-Act requires that a risk management system is created to identify, analyse, evaluate, and mitigate risks that AI systems pose to ‘health, safety and fundamental rights’.^130^ Any residual risks should be deemed ‘acceptable’, and the deployer should be provided with the required information about those risks.^131^ Outsourcing the ‘acceptability’ of ‘residual risks’ to high-risk AI providers is hardly acceptable.^132^ This approach gives ‘undue discretion’ to AI providers.^133^ Indeed, Smuha and others state that:

At the very least, we wonder why there is no obligation for the AI provider to consult with stakeholders, such as those who will be subjected to the AI system or otherwise have a legitimate interest, about which level of risk may be deemed ‘acceptable.’ ^134^

Further, systems shall be ‘designed and developed in such a way to ensure that their operation is sufficiently transparent to enable deployers to interpret the system’s output and use it appropriately’.^135^ Similarly, the AI-Act requires that AI systems be designed to be effectively overseen by natural persons.^136^ That oversight ‘shall aim to prevent or minimise the risks to health, safety or fundamental rights that may emerge when a high-risk AI system is used’.^137^ The system should be provided to the deployer so that natural persons (who have a human oversight role) are enabled ‘to remain aware of the possible tendency of automatically relying or over-relying on the output produced by a high-risk AI system (automation bias)’.^138^

This focus on oversight and transparency for the ‘deployer’ does not include the individual subjected to the system.^139^ While the AI-Act might cover ‘natural persons’ in a hospital setting, such as a doctor or manager who are the ‘deployers’, there is no direct engagement with the needs of EU citizens and residents or other individuals that come into contact with providers and deployers.^140^ More questions arise regarding the limits of oversight mechanisms for such deployers. Thus, on the interpretability of risks, the obvious question is what threshold would suffice to enable deployers to interpret outputs. Currently, the usefulness of the transparency provided may be hampered by limitations on explainability, as noted above. Even where AI systems provide explanations for their outputs, those explanations would also need to suffice from a medical standpoint if more reliance is placed on them in providing care. For example, where an AI system predicts a percentage range (such as a 40–60 per cent likelihood of a certain outcome), what would be the appropriate level of detail to share with the patient that led to that range being given?^141^

Along the same lines, one must consider whether that information would be delivered differently by a doctor advising the patient than by how the AI system presents that information.^142^ It may be a stretch too far for medical professionals to understand the reasons for outputs in every AI system. Further, the transparency requirements in the AI-Act extend beyond explainability to the system’s characteristics, capabilities, and limitations.^143^ It is unclear how the deployer would interpret and implement that information in the medical context.

Information also flows in the opposite direction. Deployers (the hospital provider or doctor) must use AI systems according to the instructions and monitor the system’s operation based on those instructions.^144^ The deployer must ensure that the natural persons assigned human oversight have the ‘necessary competence, training and authority, as well as the necessary support’.^145^ It is not clear who might be provided with such training and responsibility in the hospital setting. Nevertheless, where a natural person considers that the system poses a risk, they should inform the provider or distributor and relevant market surveillance authority and suspend the use of the system. The same is required when the deployer identifies a serious incident.^146^ Again, there is no clarity on what amounts to a ‘risk’ or a ‘serious incident’ in the medical context that would oblige a report. If the patient is using the AI system daily and cannot determine whether a system poses a ‘risk’, then it is not clear how the person assigned to oversee the system is supposed to know or how responsibility unfolds in those circumstances. If a report is not provided where there is a serious incident and the patient is harmed, the provider presumably will not be responsible. Further, how can one determine when a serious incident constitutes a breach of obligations under Union law and, therefore, meet the threshold for informing the provider or distributor?

If these questions remain unclear, then hospitals and doctors will be disempowered. The providers will determine the acceptability of risks and the threshold for providing required information. Yet, those questions should be a matter for the hospitals to have ongoing involvement in due to the evolving nature of AI. Risks with AI systems could bear on the standard of care delivered, and it should not be left entirely to the provider to make those determinations. Meanwhile, the hospital could be burdened with obligations and the possibility of liability should they fail to identify ‘risks’ with AI systems that they were not involved in determining the acceptability of. Many of these problems are exacerbated by the missing line of accountability between the provider and patient.

### D. Powerless patients?

The AI-Act has binding legal force in all EU Member States. Any rights conferred upon individuals that are clear and precise have direct effect, which means that covered individuals may invoke those rights before national courts.^147^ Nevertheless, the AI-Act emphasises obligations rather than creating new rights. Anyone seeking to challenge the deployment of an AI system for breaching the AI-Act would need to establish that they have suffered individual harm. However, it has been argued that the AI-Act must ‘do much more’ to protect the rights of consumers and be more incisive about providing measures for individuals to redress harm.^148^ In reality, the ultimate end users of AI systems are almost entirely side-lined in the regulation. While the AI-Act confers upon individuals the right to complain about an infringement of the regulation, an opportunity is lost for end users to participate in the risk mitigation aims of the AI-Act and address potential health, safety and fundamental rights concerns before they happen. The AI-Act seems to have ‘forgotten’ about ‘ordinary people’ who should have a more substantive role in consultations.^149^

Instead, patients will be restricted from enforcing their rights on the back end once a system has been deployed after they have been harmed through the proposed liability directives discussed below. For example, there are rules on data governance concerning the training, validation, and testing of datasets. Those rules cover practices concerning design choice, assessments of the quantity and quality of the data needed, and an examination of potential biases that are likely to affect health and safety or lead to discrimination.^150^ The instructions accompanying the AI system shall specify the accuracy, robustness, and cybersecurity of the system that can be expected; the circumstances in which the system may lead to risks for health and safety or fundamental rights; the performance regarding specific persons on whom the system will be used; the human oversight of the system; and the measures implemented to facilitate the interpretation of AI outputs by deployers.^151^ All these factors obscure a fundamental omission—patient input.

Even if the provisions above may be interpreted in the future to protect patient data, the problems elsewhere do not inspire confidence in creating a new regulatory system. Patients lack rights on how data is used in AI systems that may be deployed to treat them, and there are no rights in the AI-Act to protect their medical data.^152^ In particular, the AI-Act has been designed to complement the GDPR but does not compensate for its limitations.^153^ Under the GDPR, authorities sometimes lack the resources to fulfil their obligations, and the complaint mechanisms are characterized by ‘inaction and paralysis’.^154^ There are also ‘competence collisions’ between the courts on GDPR disputes.^155^ These factors raise concerns that the AI-Act will ‘play out in an even more lacklustre way than it has with the GDPR to date’.^156^ Neither regulation provides the right of patients to object to the decisions of AI systems. The AI-Act acknowledges individuals’ right to request explanations on AI’s role in the decision-making process and the decision itself, but this does not equate to a right to object.^157^ The consequences of this omission will be important to examine in the future. If AI systems become more embedded in routine care, and patients cannot object, will patients be required to seek treatment elsewhere?

Furthermore, the provisions emphasise the importance of respecting fundamental rights, yet they are unclear about where responsibility lies for respecting those rights and how they will be enforced. The AI-Act has not addressed the burden of proof for alleged breaches despite the inclusion of requirements on traceability.^158^ Causation is another significant hurdle because it will be inherently complex to prove a causal link between the decision of the AI system and the harm that is subsequently caused.^159^

Consequently, there are shortfalls in citizens’ access to justice, information, and the right to participate in public decision-making about AI systems under the AI-Act.^160^ The regulation is inadequate when benchmarked against the Commission’s commitments in previous publications to developing Ethics Guidelines for Trustworthy AI.^161^ One cannot attain legally trustworthy AI if there is no clear allocation and distribution of responsibility for wrongs and harms, which includes the protection of fundamental rights.^162^ The rule of law cannot be upheld where there is no proper framework for enforcing legal rights and responsibilities, nor where the law itself is unclear. If the ultimate user is side-lined, there cannot be full lines of accountability for the harm they suffer.^163^ Ultimately, the AI-Act reduces the careful balancing exercise required in fundamental rights to a technocratic process premised on a ‘weaker form of market-focused regulation’.^164^ Regulations should focus on specific and clear protections against fundamental rights interferences generated by AI systems.^165^

For patients, the current approach threatens to disempower them by reducing the autonomy they have gained as consumers in recent decades. The regulation sidelines them from crucial legal processes concerning developing and deploying systems that could significantly impact their care. These systems could one day usurp some of the decisions made by their doctors today. If patients are reduced to seeking redress through an opaque patchwork of disparate processes in different countries, they will be deterred.

## V. THE AILD AND THE REVISED PLD

While the AI-Act fails to address matters of liability for patients harmed by AI systems, redress for patients may be found in the proposed liability directives—the Artificial Intelligence Liability Directive (AILD) and the revised Product Liability Directive (revised PLD), published in 2022.^166^ The AILD establishes ‘fault-based liability’ for harms caused by AI systems, and the revised PLD harmonises ‘no-fault-based (strict) liability’ for product defects.^167^

### A. AILD

The existing EU liability framework comprises the PLD (discussed below) and the parallel national liability rules.^168^ National regimes might provide pathways for victims to seek compensation, but the unique characteristics of AI complicate attribution of damage to humans.^169^ Thus, the AILD seeks to harmonise non-contractual civil liability rules to make it easier for victims of AI-related damage to be compensated, ensuring individuals have the same standards of protection for harms caused under any other circumstances.^170^ It aims to achieve this through two main safeguards—the presumption of causality and the right of access to information about AI systems.

Firstly, under the current national liability regime, the burden of proof for a fault-based claim rests with the victim, who must prove damage, fault, and causality.^171^ Establishing fault may be straightforward in most doctor–patient interactions. However, AI complicates lines of responsibility, and victims often lack the costly technical expertise and analytical capacity required to understand opaque AI systems and may, therefore, struggle with establishing causation.^172^ The AILD alleviates this burden of proof by creating a ‘presumption of causality’, meaning that where victims demonstrate that someone failed to comply with a duty of care under Union or national law relevant to the harm, and a reasonably likely causal relationship with AI performance exists, the court may infer that such non-compliance caused the damage.^173^ The defendant may rebut this presumption by proving that ‘its fault could not have caused the damage’ or by demonstrating that the victim had reasonable access to ‘sufficient evidence and expertise’ to prove the causal link.^174^

Secondly, multiple actors, including software developers, manufacturers, algorithm trainers, and end users, are involved in the design, development, deployment, and operation of AI systems.^175^ Identifying the tortfeasor in this matrix is exceedingly challenging, leaving victims with the daunting task of demonstrating fault and causation, which is necessary for successful claims.^176^ The AILD provides victims access to relevant information by empowering national courts to order the disclosure of evidence regarding high-risk AI systems suspected to have caused damage.^177^ This will allow victims to identify those potentially liable for harm, such as doctors who use AI diagnostic systems without following the instructions for use.^178^

Although the AI-Act includes a similar transparency requirement directed towards the deployer of the system, the AILD goes a step further by requiring the disclosure of information to any victim of AI harm—thus empowering the end users (ie, the patients) that ultimately suffer the harm.^179^ By easing the burden of proof and granting rights to access relevant information, the AILD seeks to strengthen patient rights and autonomy within the doctor–patient relationship and potentially increase transparency and accountability in healthcare settings. The legal repercussions may prompt doctors to be more cautious when using AI, communicating to patients the recommendations of AI tools that might be used in their care, and helping foster trust by explaining how a particular decision has been made.^180^

Nevertheless, those benefits may be limited when attempting to prove fault within complex medical AI systems.^181^ Consider a black-box medical AI that produces an output the doctor reviews and relies upon but cannot independently assess for accuracy. Under the AILD, the physician relying on it will likely not be held liable for violating an applicable duty of care as they cannot understand AI’s algorithmic process and could not have known that AI’s prediction was incorrect.^182^ Identifying a breach of duty of care by any party would be challenging. This is because the AILD does not create ‘any new substantive duties of care for manufacturers or individual/organizational healthcare providers’, but instead relies on existing duties of care to assess fault.^183^ This is problematic as the inner workings of AI systems ‘cannot all be measured according to duties of care designed for human conduct’, again leaving patients with the tremendous challenge of demonstrating fault in complex AI systems.^184^ Furthermore, the AILD extensively references the AI-Act to maintain coherence between legal instruments. However, the directive’s applicability depends on concepts imported from the AI-Act, making it susceptible to the regulation’s weaknesses.^185^

Thus, while the AILD may empower patients to seek redress for AI-related harm and promote accountability through its mechanisms, the opacity of medical AI systems and excessive reliance on concepts from the AI-Act may limit its effectiveness. Additionally, the increased legal scrutiny may lead to defensive medicine, whereby doctors prioritise legal protection over patient care by exercising over-cautiousness or excessive intervention that adversely impacts treatment decisions and patient care.^186^ Conversely, this paradigm could lead to the instrumentalization of care noted above, where doctors overly rely on AI outputs because their employer hospitals require its use, and deviating from its use could fall below the standard of care required. Ultimately, the AILD’s impact on the doctor–patient relationship will depend on how healthcare providers implement AI and the corollary expectations placed on doctors.^187^

### B. Revised PLD

Alongside the AILD is the revised PLD that aims to modernise the existing EU regime on strict liability for producers of defective products.^188^ The existing PLD applies to products ranging from raw materials to cancer medicines and AI-enabled devices.^189^ It allows victims to bring claims against the producer of a defective product (and, in some cases, the supplier/seller) for a defect present when the product is placed on the market.^190^ The victims may claim compensation for death, personal injury, or material damage caused by an item or product intended for private use if they can prove the product’s defect, the damage suffered, and the causal link between the two.^191^

In its current form, the PLD falls short of regulating emerging digital technologies, and provides inadequate compensation for damages caused by AI.^192^ The PLD’s definition of ‘product’ raises ambiguities regarding its scope, potentially excluding AI systems from its coverage.^193^ The PLD’s defences for producers, such as the later-defect defence and the development risk defence, may shield them from liability for defects emerging after market introduction or due to evolving scientific knowledge.^194^ Additionally, unclear rules on modified products allow refurbishers and remanufacturers to evade liability.^195^ Proving defectiveness and establishing causality is often also challenging for evolving AI systems because of limited access to technical information and the absence of explicit provisions on the burden of proof.^196^

The revised PLD seeks to broaden the definition of ‘product’ to include software, digital manufacturing files, and digital services. It also expands the concept of ‘damage’ to cover material losses resulting from death and personal injury, including medically recognised psychological harm, property damage, and data loss.^197^ A patient can claim against a software manufacturer if, for instance, an AI medical device erroneously fails to notify the doctor when a patient has a heart attack.^198^ Moreover, while the revised PLD maintains the existing defences, in certain circumstances, a manufacturer will continue to be held liable for a defect occurring after the product is placed on the market, where such a defect is due to software updates, failure to address cybersecurity vulnerabilities, or machine learning.^199^

The revised PLD also explicitly holds ‘any economic operator who has substantially modified the product outside the manufacturer’s control liable for any defect’ and treats that party as a manufacturer.^200^ This is particularly significant within the healthcare sector, where medical devices and equipment are often refurbished. Thus, the patient can identify the tortfeasor and seek redress for damage.^201^

Manufacturers must disclose necessary information in court when the victim presents sufficient evidence supporting the plausibility of their compensation claim.^202^ It further eases the burden of proof for the victim by establishing presumptions of defectiveness and a causal link under certain conditions. For example, when the manufacturer fails to disclose information, the product does not meet safety requirements or damage caused by an obvious product malfunction. A causal link is presumed when damage aligns with the defect in question or technical complexity renders proving liability excessively difficult—thus enhancing patients’ ability to bring claims.^203^

These provisions may provide greater powers of redress to patient–consumers harmed by AI used for their care. However, as with the AILD, the revised PLD has limitations that raise concerns. An overarching concern is that the text is ambiguous, reducing the directive’s effectiveness and resulting in legal uncertainty for AI developers, healthcare providers, and patients.^204^ For instance, the revised PLD does not provide much guidance on applying the concept of a ‘defect’ to autonomous AI systems, making it impossible to differentiate between ‘harm resulting from AI’s autonomous decisions and harm resulting from a defect’.^205^ If an autonomous AI makes medical decisions without a doctor’s input, patients who are harmed may struggle to hold the manufacturer liable. The AI’s non-interpretable reasoning might be considered beyond the manufacturer’s control under the revised PLD.^206^ In such cases, patients will often require expert advice to assess the causal link, which will increase legal costs, likely discouraging them from bringing a claim.^207^

Furthermore, under the revised PLD, a product is deemed defective if ‘it does not provide the safety which the public at large is entitled to expect’.^208^ Yet, distinguishing between acceptable and unacceptable healthcare outcomes can be challenging, resulting in unclear baseline expectations and perhaps demands for minimum safety standards beyond simple programming errors.^209^ Though adequate warning about foreseeable or unavoidable risks inherent in an autonomous system may exempt manufacturers from liability in some sectors, it is unclear ‘what specific information and level of detail is necessary in these warnings’ to ensure doctors and patients comprehend the risks. ^210^

Together, the AILD and revised PLD paint a complex picture of the doctor–patient relationship. The provisions provide new and enhanced powers to patients premised on promoting greater transparency and accountability. Nevertheless, it is doubtful whether the provisions do enough to deal with the threat of the disempowerment of patients and doctors. The directives are ambiguous, the processes are complex for the average person to pursue, and the thresholds for proof may be hard to establish. Further, while designed not to overlap (the AILD targets AI specifically, whereas the revised PLD applies broadly to software), the AILD may harmonise strict liability rules for AI systems with a certain risk profile in the future, potentially blurring the lines between the directives, and exacerbating selection of the proper compensation regime for victims.^211^ Critics argue that dual directives, instead of one comprehensive regulation, contribute to fragmentation among Member States, further complicating victims’ ability to understand applicable regulations and seek redress across borders.^212^ Ultimately, the directives create complex and hard-to-attain legal thresholds that may do little to quell concerns about the disempowerment of doctors and patients to the companies that develop AI systems.

## VI. CONCLUSION

The doctor–patient relationship has evolved from one underpinned by paternalism, where doctors held the dominant role and patients followed their guidance without much explanation (‘physician as a parent’ model), to one championing the patient–consumer imbued with autonomy (models such as the ‘physician as partner’ and the ‘physician as service provider’ that emphasise patients as active participants in their healthcare decisions). Driven by court rulings and professional guidelines, this evolution reflects broader cultural trends towards the rise of individualism and market-driven healthcare dynamics, where patients are increasingly viewed as rights-holders and consumers capable of making informed choices.

Highlighting the different models of the doctor–patient relationship, this article examined the impact of technology on doctor–patient care and the resulting shift in these models, with technology providing more information and options to both healthcare professionals and patients. The integration of AI, in particular, can amplify those benefits, enhancing patient autonomy by providing access to health information and enabling new tools for patient empowerment, such as remote monitoring. However, AI’s growing sophistication presents unique challenges that threaten to erode the autonomy gained by disempowering patients and doctors alike and shifting controls to external market forces. Although AI’s potential to enhance diagnostic accuracy and support informed decision-making seems promising, it risks over-reliance by doctors, diminished personal interaction with patients, and raises concerns about data privacy, opacity, and accountability.

The EU’s primary approach to regulating AI seeks to create a risk classification system through the AI-Act. That regulation is flawed in the healthcare context with its technocratic focus and lack of clear allocation of responsibility. The Act fails to capture the nuanced nature of the doctor–patient relationship, side-lining patients (the ultimate end-users of AI systems) in the regulatory process, and may reduce their autonomy and ability to participate meaningfully in decisions that affect their care. The regulation’s reliance on self-assessments and private sector certification further raises serious concerns about the efficacy and independence of compliance mechanisms. Rather than empowering doctors and patients and upholding autonomy, the Act could inadvertently accelerate their disempowerment, leading to a decline in the role of the patient and greater prominence in the role of AI companies shielded by a veil of complex law.

The proposed AILD and the revised PLD mark important advancements in the legal landscape for AI in healthcare. By harmonising liability rules, easing the burden of proof for victims, and providing greater transparency and accountability, the proposed directives may be tools for empowerment on paper, but it is unlikely they will be wielded by patients and care providers in substantive and meaningful ways owing to their ambiguity. The dual directives may inadvertently complicate the compensation process for patients, potentially exacerbating the disempowerment of both patients and doctors in their reliance on AI systems. The law should stand ready to protect patient autonomy and ensure that healthcare professionals retain a primary role in determining risk. To do otherwise would risk a future in which private entities determine the standard of care in a vacuum that ignores those most affected by AI systems.

## Footnotes

1 Graeme Laurie, Shawn Harmon and Gerard Porter, *Mason & McCall Smith’s Law & Medical Ethics* (10th edn, OUP 2016) 9.

2 Thierry Garrot and Nathalie Angele-Halgand, ‘Digital Health Business Models: Reconciling Individual Focus and Equity’ in Loick Menvielle, Anne-Francois Audrain-Pontevia and William Menvielle, *The Digitalization of Healthcare: Challenges and Opportunities* (Palgrave MacMillian 2017) 59.

3 Carlo Botrugno, ‘Telemedicine in Daily Practice: Addressing Legal Challenges While Waiting for an EU Regulatory Framework’ (2018) 7 Health Policy and Technology 131.

4 DonHee Lee and Seong No Yoon, ‘Application of Artificial Intelligence-Based Technologies in the Healthcare Industry: Opportunities and Challenges’ (2021) 18 International Journal of Environmental Research and Public Health 271, 271; Mount Sinai, ‘Mount Sinai Launches Department of Artificial Intelligence and Human Health’ (*Mount Sinai*, 11 October 2021) <https://www.mountsinai.org/about/newsroom/2021/mount-sinai-launches-department-of-artificial-intelligence-and-human-health> accessed 3 April 2024.

5 Regulation (EU) 2024/1689 of the European Parliament and of the Council of 13 June 2024 Laying Down Harmonised Rules on Artificial Intelligence and Amending Regulations (EC) No 300/2008, (EU) No 167/2013, (EU) No 168/2013, (EU) 2018/858, (EU) 2018/1139, and (EU) 2019/2144, and Directives 2014/90/EU, (EU) 2016/797, and (EU) 2020/1828 (Artificial Intelligence Act) [2024] OJ L (AI-Act); for an overview of legal developments for AI in healthcare in the EU see, Timo Minssen and others, ‘Governing AI in the European Union: Emerging Infrastructures and Regulatory Ecosystems in Health’ in Barry Solaiman and I Glenn Cohen (eds), *Research Handbook on Health, AI and the Law* (Edward Elgar 2024); for a detailed overview of legal developments globally, see, Barry Solaiman and I Glenn Cohen (eds), *Research Handbook on Health, AI and the Law* (Edward Elgar 2024); see generally, Anwar Al-Hwsali and others, ‘Scoping Review: Legal and Ethical Principles of Artificial Intelligence in Public Health’ (2023) 305 Studies in Health Technology and Informatics 640; see, generally, Barry Solaiman, ‘From ‘AI to Law’ in Healthcare: The Proliferation of Global Guidelines in a Void of Legal Uncertainty’ (2023) 42 Medicine and Law 391.

6 Jonathan Herring, *Medical Law and Ethics* (8th edn, OUP 2020) 11.

7 Laure, Harmon and Porter (n 1) 9–10.

8 ibid.

9 Herring (n 6) 11.

10 [2015] UKSC 11[81].

11 General Medical Council, ‘The Duties of a Doctor Registered with the GMC’ (*General Medical Council*) <https://www.gmc-uk.org/professional-standards/professional-standards-for-doctors/good-medical-practice/the-duties-of-medical-professionals-registered-with-the-gmc> accessed 3 April 2024.

12 Alex Mold, ‘Making British Patients into Consumers’ (2015) 385 The Lancet 1286, 1286.

13 ibid.

14 Mani Shutzberg, ‘The Doctor as Parent, Partner, Provider… Or Comrade? Distribution of Power in Past and Present Models of the Doctor-Patient Relationship’ (2021) 29 Health Care Analysis 231, 234.

15 Barbara J Evans, ‘Big Data and Individual Autonomy in a Crowd’ in I Glenn Cohen and others (eds), *Big Data, Health Law, and Bioethics* (CUP 2018) 20.

16 ibid.

17 Alex Mold, ‘Patient Groups and the Construction of the Patient-Consumer in Britain: An Historical Overview’ (2010) 39 Journal of Social Policy 505, 507.

18 Albert van der Zeijden, ‘The Patient Rights Movement in Europe’ (2000) 18 Pharmacoeconomics 7, 8.

19 Tamara Hervey and Jean McHale, *European Union Health Law: Themes and Implications* (CUP 2015) 75, 96.

20
*Montgomery* (n 10) [75].

21 Hervey and McHale (n 19) 75; Lila Skountridaki, ‘The Patient-Doctor Relationship in the Transnational Healthcare Context’ (2019) 41 Sociology of Health and Illness 1685, 1685.

22 Shaun Pattinson, *Medical Law and Ethics* (5th edn, Sweet & Maxwell 2017) 25.

23 Skountridaki (n 21) 1686.

24 Shutzberg (n 14) 232; Brent Mittelstadt, ‘The Impact of Artificial Intelligence on the Doctor-Patient Relationship’ (Council of Europe 2021) 36.

25 Shutzberg (n 14) 232; See also, David Thomasma, ‘Beyond Medical Paternalism and Patient Autonomy: A Model of Physician Conscience for the Physician-Patient Relationship’ (1983) 98 Annals of Internal Medicine 243, 243.

26 The term ‘contractualism’ is used in, Laura Palazzani and others, *The Ethical Implications of New Health Technologies and Citizen Participation* (European Commission 2015) 35, 36.

27 Shutzberg (n 14) 233.

28 ibid 234; See also, Analee Beisecker and Thomas Beisecker, ‘Using Metaphors to Characterize Doctor-Patient Relationships: Paternalism Versus Consumerism’ (1993) 5 Health Communication 41, 49.

29 ibid.

30 Shutzberg (n 14) 234; Beisecker and Beisecker (n 28) 50.

31 Shutzberg (n 14) 234.

32 Palazzani and others (n 26) 35.

33 David Mechanic, ‘Changing Medical Organization and The Erosion of Trust’ (1996) 74 The Milbank Quarterly 171, 173; Geva Greenfield and others, ‘Patient–Physician Relationships in Second Opinion Encounters—The Physicians’ Perspective’ (2012) 75 Social Science & Medicine 1202, 1210.

34 Shutzberg (n 14) 235.

35 ibid; Indeed, healthcare in Europe has become more private. See generally, Hans Maarse, ‘The Privatization of Healthcare in Europe: An Eight-Country Analysis’ (2006) 31 Journal of Health Politics, Policy and Law 981; Sue McGregor, ‘Neoliberalism and Health Care. International Journal of Consumer Studies’ (2001) 25 International Journal of Consumer Studies 82, 83.

36 Shutzberg (n 14) 235.

37 Skountridaki (n 21) 1688.

38 Oli Williams and Simone Fullagar, ‘Lifestyle Drift and the Phenomenon of “Citizen Shift” in Contemporary UK Health Policy’ (2019) 41 Sociology of Health & Illness 20, 31.

39 Garrot and Angele-Halgand (n 2) 64.

40 Grant Cumming and others, ‘Formulating eHealth Utilizing an Ecological Understanding’ in Loick Menvielle, Anne-Francois Audrain-Pontevia and William Menvielle (eds), *The Digitalization of Healthcare: Challenges and Opportunities* (Palgrave MacMillian 2017) 82.

41 George Engel, ‘The Need for a New Medical Model: A Challenge for Biomedicine’ (1977) 196 Science 129.

42 Phil Hickey, ‘Medical Model vs. Psychosocial/Behavioral Model’ (*Behaviorism and Mental Health*, 1 August 2013) <https://www.behaviorismandmentalhealth.com/medical-model-vs-psychosocialbehavioral-model/> accessed 3 April 2024.

43 ibid.

44 Mittelstadt (n 24) 37 citing Ezekiel J Emanuel and Linda L Emanuel, ‘Four Models of the Physician-Patient Relationship’ (1992) 267 Journal of American Medical Association 2221.

45 Anne-Francoise Audrain-Pontevia, William Menvielle and Loick Menvielle, ‘Patient Communities: A New Paradigm for Medicine’ in Loick Menvielle, Anne-Francois Audrain-Pontevia and William Menvielle (eds),*The Digitalization of Healthcare: Challenges and Opportunities* (Palgrave MacMillian 2017) 226; Mittelstadt (n 24) 42.

46 Garrot and Angele-Halgand (n 2) 61–64; See, generally, Kyle Rose, ‘Mobile Health: Telemedicine’s Latest Wave but This Time It’s for Real’ in Loick Menvielle, Anne-Francoise Audrain-Pontevia and William Menvielle (eds) *The Digitalization of Healthcare: Challenges and Opportunities* (Palgrave MacMillian 2017) 155–70; Linda Eftychiou and Christo El Morr, ‘Mobile Mental Health Virtual Communities: Challenges and Opportunities’ in Loick Menvielle, Anne-Francoise Audrain-Pontevia and William Menvielle (eds), *The Digitalization of Healthcare: Challenges and Opportunities* (Palgrave MacMillian 2017) 259; on physicians using mobile phones, see Melvyn Zhang and Roger Ho, ‘M-Health and Smartphone Technologies and Their Impact on Patient Care and Empowerment’ in Loick Menvielle, Anne-Francoise Audrain-Pontevia and William Menvielle (eds), *The Digitalization of Healthcare: Challenges and Opportunities* (Palgrave MacMillian 2017) 278.

47 ‘Exploring Digital Therapeutics, Artificial Intelligence, and Virtual Coaches’ (*EHTEL*, 2020) <https://www.ehtel.eu/18-articles/67-digital-therapeutics-artificial-intelligence-and-virtual-coaches.html> accessed 33 April 2024; Barry Solaiman, ‘Addressing Access with Artificial Intelligence: Overcoming the. Limitations of Deep Learning to Broaden Remote Care Today’ (2020) 51 University of Memphis Law Review 1103, 1104; on ‘empowering patients’, see Zhang and Ho (n 46) 281–83; on ‘motivating’ individuals, see Sergio Gago-Masague, Thomas M Chen and Guann-Pyng Li, ‘Promoting Physical Exercise Through Embodied Trainers: A Systematic Literature Review’ in Loick Menvielle, Anne-Francoise Audrain-Pontevia and William Menvielle (eds) *The Digitalization of Healthcare: Challenges and Opportunities* (Palgrave MacMillian 2017) 294.

48 Solaiman (n 47).

49 Zhang and Ho (n 46) 279.

50 Glyn Elwyn and others, ‘Shared Decision Making: A Model for Clinical Practice’ (2012) 27 Journal of General Internal Medicine 1361, 1362–63.

51 Cumming and others (n 40) 84.

52 Alain Loute and Jean-Philippe Cobbaut, ‘What Ethics for Telemedicine?’ in Loick Menvielle, Anne-Francoise Audrain-Pontevia and William Menvielle (eds), *The Digitalization of Healthcare: Challenges and Opportunities* (Palgrave MacMillian 2017) 405.

53 ibid.

54 Palazzani and others (n 26) 34.

55 ibid 36.

56 ibid 34; Loute and Cobbaut (n 52) 405.

57 Palazzani and others (n 26) 36.

58 ibid 40.

59 ibid 36.

60 Mittelstadt (n 24) 51–52.

61 Palazzani and others (n 26) 36.

62 See Adam Bohr and Kaveh Memarzadeh (eds), *Artificial Intelligence in Healthcare* (Elsevier 2020); Tianhua Chen and others (eds), *Artificial Intelligence in Healthcare: Recent Applications and Developments* (1st edn, Springer Singapore 2022).

63 See generally, Brendan Kelly and others, ‘Radiology Artificial Intelligence: A Systematic Review and Evaluation of Methods (RAISE)’ (2022) 32 European Radiology 7998; David Killock, ‘AI Outperforms Radiologists in Mammographic Screening’ (2020) 17 Nature Review Clinical Oncology 134; another example is the use of AI in mental health, see, Barry Solaiman, Abeer Malik and Suhaila Ghuloum, ‘Monitoring Mental Health: Legal and Ethical Considerations of Using Artificial Intelligence in Psychiatric Wards’ (2023) 49 American Journal of Law & Medicine 250.

64 Jay Carrierand others, ‘Case Report: Utilizing AI and NLP to Assist with Healthcare and Rehabilitation During the COVID-19 Pandemic’ (2021) 4 Frontiers in Artificial Intelligence 1, 4; Young Juhn and Hongfang Liu, ‘Artificial Intelligence Approaches Using Natural Language Processing to Advance EHR-Based Clinical Research’ (2020) 14 Journal of Allergy and Clinical Immunology 463.

65 Barry Solaiman and Mark Bloom, ‘AI, Explainability, and Safeguarding Patient Safety in Europe: Toward a Science-Focused Regulatory Model’ in I Glenn Cohen and others (eds), *The Future of Medical Device Regulation: Innovation and Protection* (CUP 2022) 92; Anita Ramachandran and Anupama Karuppiah, ‘A Survey on Recent Advances in Wearable Fall Detection Systems’ (2020) BioMed Research International 1; Ahmed A and others, ‘Wearable Devices for Anxiety & Depression: A Scoping Review’ (2023) 3 Computer Methods and Programs in Biomedicine Update; see also, the use of AI remotely in the metaverse through virtual reality, Barry Solaiman, ‘Telehealth in the Metaverse: Legal & Ethical Challenges for Cross-Border Care in Virtual Worlds’ (2023) 51 Journal of Law, Medicine & Ethics 287.

66 Pranav Rakpurkar and others, ‘AI in Health and Medicine’ (2022) 28 Nature Medicine 31, 34.

67 Garrot and Angele-Halgand (n 2) 64.

68 Cumming and others (n 40) 82.

69 Shutzberg (n 14) 235.

70 Sally Dalton-Brown, ‘The Ethics of Medical AI and the Physician-Patient Relationship’ (2020) 29 Cambridge Quarterly of Healthcare Ethics 115, 118.

71 ibid.

72 ibid.

73 Mittelstadt (n 24) 42–43.

74 Dalton-Brown (n 70); Hannah Van Kolfschooten, ‘EU Regulation of Artificial Intelligence: Challenges for Patients’ Rights (2022) 59 Common Market Law Review 81, 93.

75 Rakpurkar and others (n 66) 35.

76 Mittelstadt (n 24) 55.

77 Sara Gerke, Timo Minssen and I Glenn Cohen, ‘Ethical and Legal Challenges of Artificial Intelligence-Driven Healthcare’ in Adam Bohr and Kaveh Memarzadeh (eds), *Artificial Intelligence in Healthcare* (Elsevier 2020) 305.

78 It is beyond the scope of this article to analyse the GDPR. For an analysis, see Solaiman (n 47) 1126.

79 Rakpurkar and others (n 66) 35.

80 Gerke, Minssen and Cohen (n 77) 302; Kolfschooten (n 74) 96.

81 Mittelstadt (n 24) 46.

82 ibid.

83 I Glenn Cohen, ‘Informed Consent and Medical Artificial Intelligence: What to Tell the Patient’ (2020) 108 The Georgetown Law Journal 1425, 1428.

84 Rakpurkar and others (n 66) 35.

85 W Nicholson Price II, Sara Gerke and I Glenn Cohen, ‘Liability for Use of Artificial Intelligence in Medicine’ in Barry Solaiman and I Glenn Cohen (eds), *Research Handbook on Health, AI and the Law* (Edward Elgar 2024) 154.

86 Rakpurkar and others (n 66) 35; Mittelstadt (n 24) 53.

87 Kolfschooten (n 74) 93.

88 ibid.

89 Rakpurkar and others (n 66) 35.

90 Price II, Gerke and Cohen (n 85) 19–20.

91 AI-Act (n 5).

92 Kolfschooten (n 74) 106.

93 ibid.

94 ibid.

95 Michael Veale and Frederik Zuiderveen Borgesius, ‘Demystifying the Draft EU Artificial Intelligence Act: Analysing the Good, the Bad, and the Unclear Elements of the Proposed Approach’ (2021) 4 Computer Law Review International 97, 112.

96 AI-Act (n 5) Recital 26.

97 ibid art 3(1).

98 ibid art 3(3).

99 ibid art 3(4).

100 ibid art 5(1)(a).

101 ibid ch IV.

102 ibid art 50(1).

103 Ibid art 50(3).

104 Vera Lucia Raposo, ‘Ex Machina: Preliminary Critical Assessment of the European Draft Act on Artificial Intelligence’ (2022) 30 International Journal of Law and Information Technology 88, 99.

105 AI-Act (n 5) Annex III.

106 Sam Clarke and others, ‘Submission of Feedback to the European Commission’s Proposal for a Regulation Laying Down Harmonised Rules on Artificial Intelligence’ (University of Cambridge (Leverhulme Centre for the Future of Intelligence and Centre for the Study of Existential Risk) 2021) 3.

107 Kolfschooten (n 74) 107–08.

108 ibid 108.

109 See generally, Saba Akbar, Enrico Coiera and Farah Magrabi, ‘Safety Concerns with Consumer-Facing Mobile Health Applications and Their Consequences: A Scoping Review’ (2020) 27 Journal of the American Medical Informatics Association 330.

110 Kolfschooten (n 74) 82.

111 AI-Act (n 5) art 6(1).

112 ibid art 6(1)(b).

113 ibid art 6(1)(a).

114 ibid art 7(2).

115 Natalie Smuha and others, ‘How Can the EU Achieve a Legally Trustworthy AI: A Response to the European Commission’s Proposal for an Artificial Intelligence Act’ (2021) Leads Lab University of Birmingham 1, 37; This can be seen in AI-Act (n 5) arts 17 and 43.

116 AI-Act (n 5) art 16.

117 Smuha and others (n 115) 39.

118 ibid.

119 Raposo (n 104) 98.

120 ibid.

121 AI-Act (n 5) art 34(1).

122 ibid art 31(4).

123 Veale and Borgesius (n 95) 106.

124 ibid; Jean-Pierre Galland, ‘The Difficulties of Regulating Markets and Risks in Europe through Notified Bodies’ (2017) 4 European Journal of Risk Regulation 365, 369.

125 Raposo (n 104) 98–99 citing Sebastian Klovig Skelton, ‘Europe’s Proposed AI Regulation Falls Short on Protecting Rights’ (*Computer Weekly*, 14 June 2021) <https://www.computerweekly.com/feature/Europes-proposed-AI-regulation-falls-short-on-protecting-rights> accessed 4 April 2024.

126 See Nick Paul Taylor, ‘Notified Bodies Join Chorus of Criticism of Proposed European AI Regs’ (*MedTechDrive*, 26 October 2021) <https://www.medtechdive.com/news/notified-bodies-EU-proposed-artificial-intelligence-ai-regulation/608880/> accessed 4 April 2024.

127 AI-Act (n 5) art 17(1).

128 ibid art 72(1) and (2).

129 ibid art 3(4).

130 ibid art 9(2).

131 ibid art 9(5).

132 Smuha and others (n 115) 29.

133 ibid.

134 ibid 30.

135 AI-Act (n 5) art 13(1).

136 ibid art 14(1).

137 ibid art 14(2).

138 ibid art 14(4).

139 Smuha and others (n 115) 35.

140 ibid.

141 Dalton-Brown (n 70) 117.

142 ibid.

143 AI-Act (n 5) art 13(3).

144 ibid art 26(5).

145 ibid art 26(2).

146 ibid art 26(5).

147 Luciano Floridi, ‘The European Legislation on AI: A Brief Analysis of its Philosophical Approach’ (2021) 34 Philosophy & Technology 215, 216.

148 ibid 218.

149 Smuha and others (n 115) 50.

150 AI-Act (n 5) art 10(2).

151 ibid art 13(3).

152 Kolfschooten (n 74) 93–98, 107–09.

153 ibid 108.

154 Nathalie Smuha, ‘Beyond the Individual: Governing AI’s Societal Harm’ (2021) 10(3) Internet Policy Review 1, 21; Veale and Borgesius (n 95) 111.

155 Raposo (n 104) 107.

156 Veale and Borgesius (n 95) 111.

157 AI-Act (n 5) art 86.

158 Yannick Meneceur, ‘European Commission’s AI Regulation Proposal: Between Too Much and Too Little?’ (*LinkedIn*, 23 April 2021) <https://www.linkedin.com/pulse/european-commissions-ai-regulation-proposal-between-too-meneceur/> accessed 4 April 2024.

159 ibid.

160 Smuha (n 154) 22.

161 Smuha and others (n 115) 6; High-Level Expert Group on Artificial Intelligence (AI HLEG), *Ethics Guidelines for Trustworthy AI* (European Commission 2019).

162 ibid.

163 ibid.

164 ibid 12.

165 ibid.

166 Commission, ‘Proposal for a Directive of the European Parliament and the Council on Adapting Non-Contractual Civil Liability Rules to Artificial Intelligence (AI Liability Directive)’ COM (2022) 496 final (AILD); Commission, ‘Proposal for a Directive of the European Parliament and of the Council on Liability for Defective Products’ COM (2022) 495 final (Revised PLD).

167 Tambiama Madiega, ‘Artificial Intelligence Liability Directive’ (European Parliamentary Research Service 2023) 2; Samar Nawaz, ‘The Proposed EU AI Liability Rules: Ease or Burden?’ (*European Law Blog*, 7 November 2022) <https://europeanlawblog.eu/2022/11/07/the-proposed-eu-ai-liability-rules-ease-or-burden/> accessed 29 February 2024.

168 Madiega (n 167) 2.

169 Commission, ‘Report on the Safety and Liability Implications of Artificial Intelligence, the Internet of Things and Robotics’ COM (2020) 64 final 13.

170 European Commission, ‘New Liability Rules on Products and AI to Protect Consumers and Foster Innovation’ (*European Commission*, 28 September 2022) <https://ec.europa.eu/commission/presscorner/detail/en/ip_22_5807> accessed 4 April 2024.

171 Madiega (n 167) 3.

172 COM (2020) 64 (n 169) 15.

173 AILD (n 166) art 4(1); Madiega (n 167) 6–7.

174 AILD (n 166) Recital 30 and art 4(4).

175 COM (2020) 64 (n 169) 14.

176 ibid.

177 AILD (n 166) art 3(1).

178 See Madiega (n 167) 7.

179 AI-Act (n 5) art 13; Nawaz (n 167).

180 Anto Čartolovni, Anamaria Malešević and Luka Poslon, ‘Critical Analysis of the AI Impact on the Patient–Physician Relationship: A Multi-Stakeholder Qualitative Study’ (2023) 9 Digital Health 1, 12.

181 Nawaz (n 167); Mindy Nunez Duffourc and Sara Gerke, ‘The Proposed EU Directives for AI Liability Leave Worrying Gaps Likely to Impact Medical AI’ (2023) 6 NPJ Digit Medicine 1, 1.

182 Duffourc and Gerke (n 181) 4.

183 ibid.

184 ibid; Expert Group on Liability and New Technologies—New Technologies Formation, *Liability for Artificial Intelligence and Other Emerging Digital Technologies* (European Union 2019) 23.

185 Philipp Hacker, ‘The European AI Liability Directives—Critique of a Half-Hearted Approach and Lessons for the Future’ (2023) 51 Computer Law & Security Review 1, 8.

186 See eg, Dane Bottomley and Donrich Thaldar, ‘Liability for Harm Caused by AI in Healthcare: An Overview of the Core Legal Concepts’ (2023) 14 Frontiers in Pharmacology 1, 5.

187 See Mittelstadt (n 24) 55, 64.

188 Stefano De Luca, ‘New Product Liability Directive’ (European Parliamentary Research Service 2023) 3.

189 European Commission, ‘Commission Staff Working Document Impact Assessment Report Accompanying the Document: Proposal for a Directive of the European Parliament and of the Council on Liability for Defective Products’ SWD (2022) 316 final 9.

190 European Commission, ‘Commission Staff Working Document Impact Assessment Report Accompanying the Document: Proposal for a Directive of the European Parliament and of the Council on Adapting Non-contractual Civil Liability Rules to Artificial Intelligence’ SWD (2022) 319 final 4.

191 De Luca (n 188) 2.

192 See Sadie Whittam, ‘Mind the Compensation Gap: Towards a New European Regime Addressing Civil Liability in the Age of AI’ (2022) 30 International Journal of Law and Information Technology 249.

193 Expert Group on Liability and New Technologies—New Technologies Formation (n 184) 28.

194 Council Directive 85/374/EEC of 25 July 1985 on the Approximation of the Laws, Regulations and Administrative Provisions of the Member States Concerning Liability for Defective Products [1985] OJ L210, art 7(b) and (e).

195 SWD (2022) 316 (n 189) 12.

196 ibid 19–20.

197 De Luca (n 188) 4–6.

198 Claudia Nicastro, ‘The EU AI Liability Directive and Product Liability Directive’ (Health Action International 2023) 2; SWD (2022) 316 (n 189) 15–16.

199 De Luca (n 188) 6; Revised PLD (n 166) art 10(2).

200 De Luca (n 188) 5; Revised PLD (n 166) art 7(4).

201 SWD (2022) 316 (n 189) 17, 70.

202 Revised PLD (n 166) art 8(1).

203 ibid Recital 34 and art 9; De Luca (n 188) 6.

204 See Shu Li and Béatrice Schütte, ‘The Proposal for a Revised Product Liability Directive: The Emperor’s New Clothes?’ (2023) Maastricht Journal of European and Comparative Law 1, 22.

205 Miriam Buiten, Alexandre de Streel and Martin Peitz, ‘The Law and Economics of AI Liability’ (2023) 14 Computer Law & Security Review 1, 15.

206 Duffourc and Gerke (n 181) 4.

207 Buiten, Streel and Peitz (n 205) 16–17.

208 Revised PLD (n 166) art 6(1).

209 Yiannos S. Tolias, ‘AI in Neurosurgery: Liability Implications in the Light of the EU Product Liability Directive Proposal’ (2023) 38 Journal du Droit de la Santé et de l’Assurance—Maladie (JDSAM) 81, 83.

210 ibid 84.

211 Hacker (n 185) 6; Li and Schütte (n 204) 23; AILD (n 166) 9.

212 Hacker (n 185) 8.

